# Molecular Mechanisms of Fibrosis in Cholestatic Liver Diseases and Regenerative Medicine-Based Therapies

**DOI:** 10.3390/cells13231997

**Published:** 2024-12-03

**Authors:** Wei-Lu Wang, Haoran Lian, Yingyu Liang, Yongqin Ye, Paul Kwong Hang Tam, Yan Chen

**Affiliations:** 1 School of Pharmacy, Faculty of Medicine, Macau University of Science and Technology, Macao SAR, China; 3220006902@student.must.edu.mo (W.-L.W.); 2230028591@student.must.edu.mo (H.L.); 3230006552@student.must.edu.mo (Y.L.); 2 Faculty of Medicine, Macau University of Science and Technology, Macao SAR, China; 2202853ghp30001@student.must.edu.mo; 3 Precision Regenerative Medicine Research Centre, Medical Sciences Division, Macau University of Science and Technology, Macao SAR, China

**Keywords:** cholestatic liver fibrosis, PBC, PSC, BA, liver transplantation, mechanism, regenerative medicine

## Abstract

The aim of this review is to explore the potential of new regenerative medicine approaches in the treatment of cholestatic liver fibrosis. Cholestatic liver diseases, such as primary biliary cholangitis (PBC), primary sclerosing cholangitis (PSC), and biliary atresia (BA), due to the accumulation of bile, often progress to liver fibrosis, cirrhosis, and liver failure. When the disease becomes severe enough to require liver transplantation. Deeply understanding the disease’s progression and fibrosis formation is crucial for better diagnosis and treatment. Current liver fibrosis treatments mainly target the root causes and no direct treatment method in fibrosis itself. Recent advances in regenerative medicine offer a potential approach that may help find the ways to target fibrosis directly, offering hope for improved outcomes. We also summarize, analyze, and discuss the current state and benefits of regenerative medicine therapies such as mesenchymal stem cell (MSC) therapy, induced pluripotent stem cells (iPSCs), and organoid technology, which may help the treatment of cholestatic liver diseases. Focusing on the latest research may reveal new targets and enhance therapeutic efficacy, potentially leading to more effective management and even curative strategies for cholestatic liver diseases.

## 1. Introduction

Liver fibrosis is a complex and dynamic process involving molecular, cellular, and tissue-level interactions that lead to the excessive buildup of extracellular matrix (ECM) components [1]. Liver fibrosis significantly contributes to global morbidity and mortality, with various causes including cholestasis [2]. Current liver fibrosis treatments mainly target the root causes and there is now only one direct treatment medicine for fibrosis [3,4], Resmetirom. The US FDA recently approved the oral method of Resmetirom for treating nonalcoholic steatohepatitis (NASH) with liver fibrosis. Its advantage lies in the fact that it outperformed a placebo at week 52 in both primary histological endpoints (NASH resolution without worsening of fibrosis, and improvement in fibrosis by ≥1 stage without worsening of nonalcoholic fatty liver disease (NAFLD) activity score) [4]. However, the long-term safety of Resmetirom remains a concern. Some other therapies or medications may represent promising tools for addressing liver fibrosis, but have not been approved for clinical treatment despite being used in human studies. Without active intervention, liver fibrosis can lead to cirrhosis, liver failure, and liver cancer [5,6].

Cholestatic liver disease affects both adults and children, but the proportion varies between these age groups. This might be attributed to differences in liver or bile duct development, the body’s response to pathogens, and environmental and lifestyle factors. Common environmental factors may include long-term exposure to certain chemicals such as industrial solvents or environmental toxins [7], while lifestyle factors may include unhealthy eating habits, excessive drinking, and a lack of adequate physical activity [8]. Additionally, long-term use of certain medications, such as hormonal drugs and antiepileptic drugs, may increase the risk of disease [9]. Prolonged exposure to these substances, especially without proper protection, can lead to and exacerbate liver dysfunction and cholestasis [7]. Furthermore, in adults, the bile duct system is fully developed, and cholestasis is usually associated with chronic diseases or long-term exposure to harmful substances. Additionally, the immune system in adults is more mature, making them more susceptible to autoimmune diseases such as primary biliary cholangitis (PBC) and primary sclerosing cholangitis (PSC) [10,11]. Compared to adults, the bile duct system in children is not fully developed and is more susceptible to genetic and congenital factors. Additionally, the bile duct system in newborns and infants is more fragile, making it prone to cholestasis due to infections and metabolic disorders. Interestingly, the incidence of different cholestatic liver diseases varies by gender, which may be due to differences in the immune system, hormone levels, and environmental and lifestyle factors between males and females [12,13]; for example, it has been reported that the proportion of men who drink alcohol is indeed higher than that of women in the United States [8]. Cholestatic liver diseases may reach the stage of liver fibrosis if they continue to develop. As a protective mechanism of the body, liver fibrosis involves tissue repair and liver regeneration in the early stage of fibrosis. In the advanced stage of fibrosis, it progresses to cirrhosis, which is irreversible and requires a liver transplant.

Liver transplantation is an effective treatment for advanced liver disease and liver failure, especially for patients whose conditions cannot be controlled by other treatments [14]. Although liver transplantation can markedly enhance the quality of life for patients with severe conditions, it remains a complex and high-risk procedure due to limitations such as donor shortages, high costs, and the necessity for long-term use of immunosuppressive drugs to prevent organ rejection. Additionally, the transplanted liver may still be susceptible to infection or damage [15,16]. Therefore, finding highly efficient and safe methods for treating fibrosis has always been crucial. Regenerative medicine, an emerging field, holds significant promise for treating cholestatic liver fibrosis. This field offers innovative approaches to managing cholestatic liver disease and fibrosis by utilizing advanced technologies such as stem cell biology, artificial organs, biomaterials, and tissue engineering. Given the current limitations of liver transplantation, regenerative medicine presents new hope and possibilities, particularly in addressing donor shortages and post-transplant complications. The primary goal of regenerative medicine is to tackle various diseases by repairing, replacing, or regenerating damaged tissues and organs. These approaches provide a beacon of hope for curing liver fibrosis.

Deeply understanding the cholestatic liver disease’s progression and fibrosis formation is crucial for better treatment. In this review, we focus on the fibrosis progression of cholestatic liver diseases such as PBC, PSC, and biliary atresia (BA), and summarize, analyze, and discuss the current landscape and advantages of regenerative medicine therapies. Our review comprehensively explores the molecular and cellular mechanisms of fibrosis in cholestatic liver diseases such as PBC, PSC, and BA. Although fibrosis is a widely studied field, our review uniquely integrates the latest advances in regenerative medicine, emphasizing innovative therapeutic strategies that target the root causes of fibrosis rather than merely managing symptoms. Our review highlights emerging therapies such as mesenchymal stem cell (MSC) therapy, induced pluripotent stem cells (iPSCs), and organoid technology, offering potential breakthroughs in managing cholestatic liver diseases. By focusing on mechanistic pathways and novel treatments, this review provides a forward-looking perspective on bridging the gap between basic science and clinical applications in liver fibrosis. This focus on the latest research may unveil new targets and enhance therapeutic efficacy, potentially leading to more effective management and even curative strategies for cholestatic liver diseases.

## 2. Bile Acid, Bile Acid Transporters, and Cholestatic Hepatocyte Injury

Bile is an important digestive fluid secreted by liver cells, consisting mainly of water, bile salts, bilirubin, cholesterol, lecithin, and inorganic salts such as sodium, potassium, and calcium [17]. Bile contains a large amount of bile acids, which are essential for maintaining normal lipid metabolism and digestive functions [18]. Bile acid transporters are key components of the enterohepatic circulation of bile acids, and their dysfunction, such as mutations in bile acid transporter genes, can lead to abnormal bile acid metabolism and subsequent cholestasis [19]. In humans, the main bile acid transporters include the apical sodium-dependent bile acid transporter (ASBT) and Na (+)-taurocholate co-transporting polypeptide (NTCP), which are expressed in the intestine and liver, respectively, and responsible for the absorption and circulation of bile acids [20]. Additionally, other transporters, like the nuclear farnesoid X receptor (FXR), plays a pivotal role in maintaining bile acid homeostasis by regulating key genes involved in bile acid synthesis, metabolism, and transport, including cholesterol 7α-hydroxylase (*CYP7A1*), bile salt export pump (*BSEP*), multidrug resistance protein 3 (*MDR3*), *ASBT*, and *NTCP* [20]. Therefore, the normal function of these transporters is crucial for maintaining the homeostasis of bile acids and liver health. When these transporters are impaired, bile acids accumulate in the bile ducts, leading to cholestasis. In the study of cholestasis, the roles of growth factors, cytokines, and chemokines are increasingly receiving attention. For example, the cholangiocyte-secreted cytokines (also termed cholangiokines) drive ductular cell proliferation, portal inflammation and fibrosis, and carcinogenesis by modulating the intrahepatic microenvironment [21]. The dynamic expression of yes-associated protein (YAP) in non-parenchymal liver cells regulates intercellular communication, which may influence inflammatory responses through TEA domain transcription factor (TEAD)-dependent transcriptional regulation [22].

High concentrations of bile acids are directly toxic to liver cells, damaging cell membranes, triggering inflammatory responses, and even causing cell death [23,24]. Furthermore, the accumulation of bile acids may inhibit cellular autophagy, affecting normal cellular metabolism and repair functions, thereby exacerbating liver damage [23]. Studies have shown that bile acids can induce liver cell death by affecting lysosomal signaling pathways, redox balance, and endoplasmic reticulum stress [25]. For example, hydrophobic bile acids may induce liver cell damage and death through mechanisms such as inducing oxidative stress responses, disrupting mitochondrial function and endoplasmic reticulum stress, activating the production of pro-inflammatory mediators, and activating death receptors [25]. These mechanisms are particularly important in cholestatic liver diseases, as they play a key role in the process of liver injury. Understanding these mechanisms is crucial for developing therapeutic strategies to mitigate bile acid-induced liver damage [26].

## 3. The Fibrotic Process in Cholestatic Liver Disease and Current Treatment

### 3.1. Fibrotic Process of PBC and Current Treatment

Primary biliary cholangitis (PBC), previously known as primary biliary cirrhosis, is a chronic autoimmune cholestatic liver disease that predominantly affects women [27]. Recent studies indicate that the global annual incidence of PBC is about 1.76 cases per 100,000 people [28]. In China, the incidence and prevalence of PBC is about 19.1 cases per 100,000 individuals [28]. The affected site is the intrahepatic bile ducts. Its characteristics include progressive damage to biliary epithelial cells (BECs), increased portal vein inflammation, and fibrosis [29,30]. The etiology of PBC includes genetic susceptibility and environmental factors, but it is not fully understood [29,31,32,33]. In the context of genetic and environmental risk factors for disease, T lymphocytes, which are supposed to protect the body from external bacterial and viral attacks, can instead begin to attack the small bile ducts arranged in a single layer by BECs within the liver lobules [34,35]. BECs express various molecular transport proteins, aquaporins, and ion channels that play a role in modifying the final composition and volume of bile [7]. When T lymphocytes attack BECs, some of these cells are destroyed, resulting in obstructed bile acid excretion and circulation. This disruption can cause bile to leak from the bile ducts into the blood or other liver cells, leading to cholestasis and potentially resulting in cholangitis [36]. Persistent inflammation may cause senescence and the formation of apoptotic bodies in BECs, ultimately leading to the irreversible loss of bile ducts. While BEC apoptosis can help clear harmful substances and promote autoimmunity [37,38], the inefficient phagocytosis of dead cells can activate the immune system. Defects in clearing dead BECs after apoptosis may specifically damage the small bile ducts in PBC [34]. Moreover, the chronic recruitment of immune cells may exacerbate the chronic inflammatory infiltration of BECs, leading to progressive bile duct damage [34,39]. The infiltration of mononuclear cells around hepatic bile duct epithelial cells can lead to degeneration and necrosis, resulting in chronic non-suppurative destructive cholangitis and the eventual destruction of medium and small bile ducts [40]. This persistent damage can continue to progress even after the removal of external triggers, such as viruses from resolved infections.

In the context of biliary tract injury, the complex relationship between cellular mechanisms and immune responses is a critical and intricate area of research. The mitochondrial pyruvate dehydrogenase complex (PDC-E2) is a key player in this process. Disruption of the bicarbonate umbrella, regulated by anion exchanger 2 (AE2), sets off a chain of events that compromise the integrity of BECs. The loss of AE2 function not only changes intracellular pH but also enhances the susceptibility of BECs to harmful bile acids, leading to apoptosis and the release of altered PDC-E2 subunits, triggering a broad immune response. The presence of anti-mitochondrial antibodies (AMAs) targeting PDC-E2 epitopes exacerbates BEC injury. This immune-driven assault involves an imbalance in effector and regulatory pathways, with effector T cells and natural killer (NK) cells significantly contributing to biliary tract damage. Understanding these mechanisms is essential for developing therapeutic approaches to manage conditions such as PBC, where immune dysregulation is a defining characteristic [41]. In PBC, the regulatory effects of circulating and intrahepatic regulatory T cells (Tregs) and T follicular regulatory T cells (TFRs) are insufficient in suppressing the injury response, resulting in persistent inflammation that exacerbates biliary tract damage [42,43].

In addition, in the early stages of PBC, macrophage aggregates form in the portal vein. The recognition of the autoantigen-AMA complex by macrophages may disrupt the liver’s immune tolerance, leading to the recruitment of various inflammatory cells into the liver [44,45,46]. APCs can subsequently trigger autoreactive T cells to secrete diverse pathological factors, thereby sustaining chronic liver inflammation and causing direct bile duct injury. Apoptotic BECs can transport immunologically intact PDC-E2 into apoptotic bodies, which, when combined with AMA and macrophages, stimulate macrophages to release potent pro-inflammatory cytokines [44,45,46], causing bile duct damage. It is worth noting that AMAs target the PDC-E2, which is located on the inner membrane of mitochondria. This may involve four processes [47]: ① Apoptosis of BECs: In PBC, the apoptosis (programmed cell death) of small BECs releases PDC-E2 into the extracellular environment. ② Modification by bile acids: Bile acids can chemically modify the lipoyl domain of PDC-E2, altering its structure and making it a neoantigen. ③ Immune Recognition: The modified PDC-E2 is then recognized as a foreign antigen by autoreactive B lymphocytes, which produce AMAs. ④ Stimulation of T Cells: This recognition stimulates T cell subpopulations, leading to an immune response and the formation of specific AMAs. This also suggests that macrophages initiate and continue the progression of PBC, but the complete mechanism remains to be studied. For example, the role of bone marrow-derived macrophages in PBC remains unknown, but may be important. Other cells, such as liver sinusoidal endothelial cells (LSECs), also play a crucial role in maintaining liver health and function. They not only act as a barrier between the portal venous blood flow and hepatocytes but also influence the immune homeostasis of the liver by regulating the flow and metabolism of bile acids. For instance, bile acids can stimulate LSECs to produce CXCL16, a molecule that attracts immune cells to the liver, aiding in tumor resistance [48]. Additionally, dysfunction of LSECs can lead to pathological processes within the liver such as inflammation, microvascular thrombosis, fibrosis, and portal hypertension [49]. A report showed that adipocyte fatty acid binding protein promotes the onset and progression of bile duct ligation (BDL)-induced cholestatic liver fibrosis via mediating the crosstalk between LSECs and hepatic stellate cells (HSCs) [50]. However, the data on LSECs related to cholestatic diseases are indeed limited.

The pathophysiology of PBC is indeed complex, involving a multifaceted interplay between cellular components and biochemical pathways. The role of BECs in PBC progression is well established, but hepatocyte dysfunction also contributes significantly to the disease [26]. FXR is a key regulator in this process. When hepatocytes are damaged, the downregulation of FXR leads to a cascade of events that exacerbate biliary injury [51]. In physiological conditions, as a sensor of bile acid, the dynamic activation of FXR is regulated by the level of intracellular bile acid, by which high levels of bile acid increase FXR expression to inhibit the biosynthesis of the bile acid or vice versa. But in pathological conditions, such as cholestasis, the accumulation of bile acid, especially the toxic intermediate and/or inflammatory cytokines, for example, interleukin 6 (IL-6), regulates FXR via signal transducer and activator of transcription 3 (STAT3) activation [52], or reactive oxygen species (ROS) largely affect the expression of FXR [51]. Furthermore, the downregulation of FXR triggers the expression of pro-inflammatory transcription factors and cytokines, such as adaptor protein-1 (AP1) and IL-6, contributing to the chronic inflammation characteristic of early PBC [26]. The resulting biliary injury is a complex response involving cellular damage, immune responses, and physiological changes. This complexity contributes to the heterogeneity in patient responses to the disease and its therapeutic interventions. Current efforts to quantify the disease burden and characterize patient heterogeneity are primarily based on clinical observations and biochemical markers such as serum alkaline phosphatase (ALP) levels and bilirubin concentrations. These markers help assess individual risk, but they do not capture the full spectrum of the disease’s dynamics. Understanding the underlying mechanisms that contribute to high-risk disease onset and progression is crucial for developing more effective treatments and personalized risk assessments. Despite the challenges, ongoing research continues to unravel the intricate mechanisms at play in PBC, aiming to improve patient outcomes through tailored therapeutic strategies [35].

After biliary tract injury, bile cannot be excreted normally and accumulates in the liver. This accumulation of bile further causes damage to the liver and bile duct cells, triggering an inflammatory response. This leads to the activation of macrophages, and the chemotactic action of necrotic cell fragments and cytokines induces the aggregation and phagocytosis of HSCs and liver macrophages. This further induces oxidative stress responses, promoting the secretion of type I and III collagen, the release of ROS and transforming growth factor-beta1 (TGF-β1), and the activation of quiescent HSCs into myofibroblasts [53]. Activated myofibroblasts secrete various cytokines, including platelet-derived growth factor (PDGF) and ECM proteins, to produce fibrous scars [54]. When bile stasis subsides [55], the activated myofibroblasts disappear [56], the inflammatory response is deactivated, or anti-inflammatory pathways are induced [57,58]. The ductular reaction (DR) promotes the healing of the wound surface around the hepatic tubules with fibrosis and inflammatory cell recruitment [59], and through Slit2-Robo1 signaling, it promotes intrahepatic angiogenesis, ECM degradation [60], and fibrous scar absorptions [61]. It is worth noting that the DR begins in the early stages of liver fibrosis progression [62], and is a reparative response of the liver following injury. This process involves the activation and proliferation of Epithelial cell adhesion molecule (EpCAM^+^) hepatic progenitor cells, which are crucial for the regeneration of hepatocytes and cholangiocytes [63,64]. During liver injury, such as damage or death of BECs, the DR promotes liver regeneration and repair by activating hepatic progenitor cells [65].

**Figure 1:**
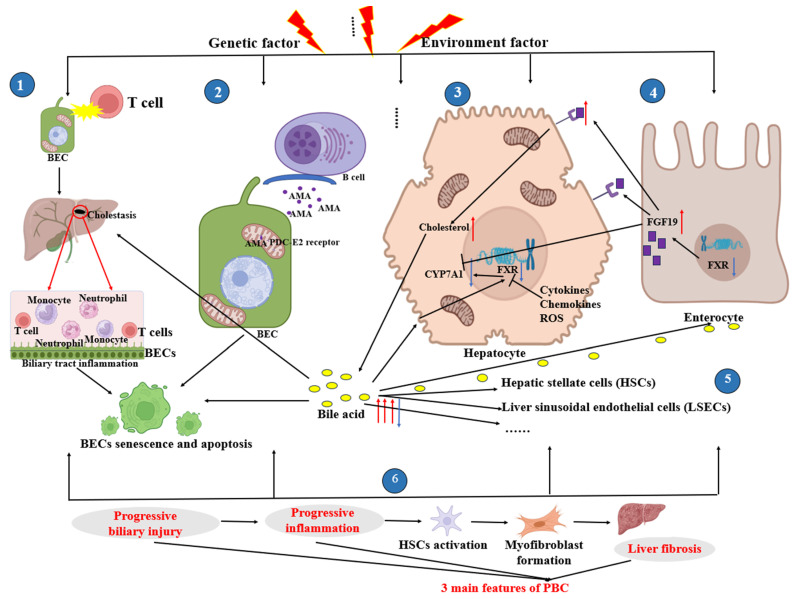
The fibrotic process of PBC. Diagrams were created with the help of BioRender software (© 2024 BioRender). The diagram displays six processes. ① Against a backdrop of genetic and environmental risk factors for disease, T lymphocytes, which should protect the body from external bacterial and viral attacks, begin to attack the small bile ducts lined by a single layer of BECs in the liver lobules, leading to bile stasis and cholangitis, as well as BEC senescence and apoptosis. ② Plasma cells secrete AMAs, which specifically recognize the PDC-E2 receptor, causing BEC damage and apoptosis. ③ and ④ When hepatocytes are damaged, especially the toxic intermediate and/or inflammatory cytokines, for example, IL-6 regulates FXR via signal transducer and activator of transcription 3 (STAT3) activation, or reactive oxygen species (ROS) largely affect the expression of FXR, with the reduction of FXR being observed. SHP induced by FXR inhibits the downregulation of CYP7A1 expression, promoting the synthesis of bile acids from cholesterol. After entering the bile ducts, the bile acids are reabsorbed into the intestinal cells via ASBT, activating the intestinal FXR, which increases the expression of FGF19. FGF19 crosses the portal circulation and subsequently binds to its receptor FGFR4/βklotho, promoting the synthesis of bile acids from cholesterol, and the excess bile acids continue to damage the bile ducts. At the same time, the increase in FGF19 may help suppress the expression of CYP7A1. However, during PBC, the expression of CYP7A1 is inhibited, which may be an adaptive response of the body to cholestatic liver injury [51]. ⑤ Bile acids act directly on hepatocytes, hepatic stellate cells (HSCs), liver sinusoidal endothelial cells (LSECs), and other cells. ⑥ Progressive biliary injury, progressive inflammation, and liver fibrosis are the three main features of PBC. A red arrow indicates an increase, a blue arrow indicates a decrease, and a black arrow indicates an effect on or causes. BECs: biliary epithelial cells; CYP7A1: cholesterol 7α-hydroxylase; FXR: farnesoid X receptor; ROS: reactive oxygen species; PBC: primary biliary cholangitis; FGF19: fibroblast growth factor 19; SHP: Small Heterodimer Partner; FGFR4/βklotho: Fibroblast Growth Factor Receptor 4/beta-Klotho.

When damaging and repairing lose their dynamic balance, prolonged inflammation leads to the replacement of healthy liver parenchyma with fibrotic tissue and regenerative nodules, resulting in portal hypertension. Liver fibrosis gradually progresses to cirrhosis, the complications of which often lead to hospitalization, an impaired quality of life, and high mortality. Progressive portal hypertension, systemic inflammation, and liver failure drive the outcome of liver transplantation [66]. The fibrotic process of PBC is summarized in Figure 1.

Currently, the first-line treatment for PBC is ursodeoxycholic acid (UDCA). It alleviates disease progression by reducing bile acid toxicity and improving bile flow, significantly enhancing patient prognosis [67,68]. However, UDCA has severe side effects, including diarrhea, weight gain, rash, and worsening pruritus [69]. Additionally, the treatment population for UDCA is limited, with approximately 40% of patients progressing to cirrhosis even after treatment [70]. Long-term use is required for UDCA, and the disease may relapse after discontinuation. When the response to UDCA is inadequate, the FXR agonist obeticholic acid (OCA) can be used as a second-line treatment to further reduce bile acid toxicity and inflammatory responses [68,69,71]. However, OCA still has serious side effects and is expensive, making it unsuitable for all patients. Fibrate drugs can be an alternative for those who do not respond well to UDCA and OCA treatment [72]. Of course, managing the symptoms of PBC is also an important part of the treatment [71]. When the condition of patients with PBC progresses to end-stage liver disease or severe complications, liver transplantation becomes an important treatment option [73].

### 3.2. Fibrotic Process of PSC and Current Treatment

Primary sclerosing cholangitis (PSC) is a complex autoimmune disease affecting the bile ducts of the liver, leading to inflammation and fibrosis [10]. The affected sites are intrahepatic and/or extrahepatic bile ducts. Recent studies indicate that the global annual incidence of PSC is about 0.6 cases per 100,000 people [74]. In China, the highest prevalence of PSC is in East China at 4.87 (95% CI: 3.44, 7.18) per 100,000, followed by North China at 2.94 (95% CI: 2.33, 3.74) per 100,000, and the lowest is in South China at 0.92 (95% CI: 0.66, 1.30) per 100,000 [75]. This condition is more common in young males and is often associated with inflammatory bowel disease (IBD), particularly ulcerative colitis (UC), and to a lesser extent, Crohn’s disease (CD) [76]. Studies have shown a significant overlap between IBD and PSC, with a large number of IBD patients developing PSC. In 2021, Brigida et al. [77] conducted an analysis on the association between IBD and PSC, revealing that among 776,700 IBD patients studied, the pooled prevalence of PSC was 2.16%, with UC and CD patients having a pooled prevalence of 2.47% and 0.9%, respectively. The prevalence was the highest in South America and the lowest in Southeast Asia. IBD is considered to be a significant risk factor for the development of PSC [78]. Approximately 60% to 80% of patients with PSC have IBD (predominantly UC in approximately 80% and CD in 20%), and approximately 5% to 10% of patients with UC have coexisting PSC [79,80]. The prevalence of PSC is higher in males than in females and varies across regions, which reflects the multifactorial etiology of PSC, where genetic susceptibility and environmental factors both contribute to its development. Besides genetic and environmental risk factors, individual background diseases also play a significant role in the risk of developing PSC. Interestingly, environmental factors may play a more significant role than genetic factors in the risk of developing PSC [79]. The association between PSC and the X chromosome also highlights the potential impact of sex-related genetic factors on the immune system and the development of autoimmune diseases. Understanding the interplay between these factors is crucial for developing targeted treatments and improving patient prognosis.

In the context of IBD, dysbiosis, chronic mucosal inflammation, and disruption of the intestinal epithelial barrier integrity are frequently observed. Microbes and microbial toxins can translate to distant sites. Bacteria translocation and the migration of pathogen-associated molecular patterns (PAMPs) can enter the portal vein circulation and reach the liver [81]. The translocation of bacteria and abnormal transport of gut lymphocytes activate innate and adaptive immune responses, further activating the liver’s immunity. In essence, antigens originating from the gut act as potential triggers, with antigen-presenting cells (APCs) carrying human leukocyte antigens (HLAs) presenting antigens to T cell receptors (TCRs), and the activated T cells may migrate to the liver and gut after clonal expansion, due to the overlapping adhesion molecule profiles of the gut and liver endothelium, such as mucosal addressin cell adhesion molecule-1 (MadCAM-1) and vascular cellular adhesion molecule-1 (VCAM-1) [82]. Anti-neutrophil cytoplasmic antibodies (ANCAs) are often observed in PSC, possibly reflecting the B cell response to gut-derived antigens [83]. At this point, the liver is affected by the shared metabolism of the gut microbes; the homeostasis of the HCO_3−_ protective umbrella of the bile duct cells is disrupted, turning bile into toxic bile; and bile acid disorder further activates BECs, causing BEC dysfunction, as well as leading to the downregulation of proteins such as takeda G protein-coupled receptor 5 (TGR5) (GPBAR1) and fatty acid regulator (FAR) in BECs [84,85], which result in bile stasis and the upregulation of proteins such as C-C motif chemokine ligand 24 (CCL24) and prolyl-4-hydroxylase alpha subunit 2 (P4HA2) [86,87], thus perpetuating inflammatory responses and cellular senescence. Peribiliary glands expand, and mesenchymal cells acquire a myofibroblast phenotype, leading to enlarged bile ducts [76]. Several immune cells have been found near the bile ducts in PSC, most notably T cells, macrophages, and neutrophils. Recent studies have reported that osteopontin is a characteristic of bile duct-associated macrophages and is associated with the severity of liver fibrosis in PSC [88]. Periportal vein macrophages prevent symbiosis driven liver inflammation [89]. A previous study found that, in PSC patients, CD68^+^CD206^+^ macrophages in the liver were increased. Additionally, the CD68^+^CD206^+^ macrophage subpopulation was associated with a significantly increased expression of TGR5 in PSC [90]. Clearly, in this study, the increase in CD68^+^CD206^+^ macrophages is associated with a pro-inflammatory phenotype [90]. Typically, in cholestatic liver disease, M1 macrophages exhibit pro-inflammatory characteristics, participating in inflammatory responses and tissue damage processes, while M2 macrophages display reparative properties, contributing to tissue repair and alleviation of inflammation [91,92]. Research has found increased peribiliary pro-inflammatory (M1-like) and alternatively activated (M2-like) monocyte-derived macrophages in PSC compared to normal livers [91]. Therefore, M1 and M2 macrophages play different but crucial roles in cholestatic liver disease. These results reflect the potential role of macrophages in PSC. The exact mechanisms involved in each cell type are not fully understood; however, crucially, they may interact and crosstalk with the active phenotype of bile duct cells.

**Figure 2:**
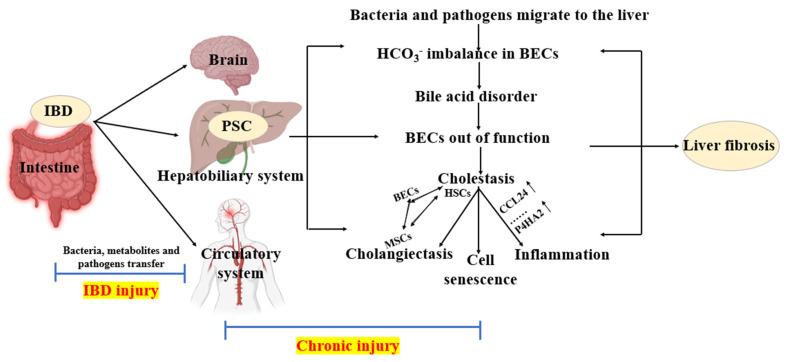
The process of IBD developing into PSC and liver fibrosis. In the context of IBD, there is translocation of bacteria, metabolites, and pathogens to the brain, liver–biliary system, and circulatory system, causing damage associated with IBD. When these agents reach the hepatobiliary system, they lead to chronic injury, resulting in PSC. At this stage, the imbalance of bicarbonate (HCO_3−_) in BECs disrupts bile acid metabolism, impairs BEC function, and leads to cholestasis. Upregulated proteins such as CCL24 and P4HA2 perpetuate inflammatory responses and cellular aging. Peribiliary glands expand, and mutual transformation occurs between BECs, MSCs, and HSCs. If chronic injury persists, it eventually leads to liver fibrosis. BECs: biliary epithelial cells; HSCs: hepatic stellate cells; MSCs: myofibroblasts. Diagrams were created with the help of BioRender software (© 2024 BioRender).

The intricate interplay between various cell types and molecular signals in the liver orchestrates the progression of cholestatic liver diseases. HSCs, portal vein myofibroblasts, and cholangiocytes interact in a complex manner following biliary dilatation, leading to chronic liver injury. The role of cholangiocyte-derived exosomal lncRNA-H19 is particularly noteworthy, as it not only promotes the generation of myofibroblasts but also contributes to the differentiation and activation of HSCs, thereby accelerating fibrosis [93]. Furthermore, the uptake of exosomal H19 by Kupffer cells—liver-specific macrophages—triggers their activation and chemotaxis via the chemokine ligand 2/chemokine receptor 2 (CCL-2/CCR-2) signaling pathway, highlighting potential therapeutic targets [94]. The upregulation of proteins such as p16, miR-200b, fibroblast growth factor 1 (FGF1), and miR-16 has been associated with the advancement of fibrosis [95,96,97], suggesting that their downregulation could thwart the fibrotic process and potentially eradicate PSC. PSC-associated complications, including bile duct stenosis, cirrhosis, and cholangiocarcinoma, often necessitate liver transplantation once cirrhosis has set in, underscoring the critical need for early intervention and novel therapeutic strategies. The main process of IBD developing into PSC and liver fibrosis is summarized in Figure 2.

However, a number of bacteria, PAMPs, and metabolites enter the systemic circulation through the liver. Meanwhile, many translocated bacteria and PAMPs enter the lymphatic vascular system from the gut, where they first pass through the mesenteric lymph nodes (MLNs). Some of these lymphatic endotoxins also enter the systemic circulation. Gut-derived bacteria, PAMPs, toxins, and metabolites can subsequently affect the function of organs such as the heart, kidneys, and brain, leading to systemic symptoms such as fatigue and itching [81]. It is important to note that many patients with PSC may not exhibit symptoms in the early stages of the disease; hence, regular check-ups are crucial for early diagnosis and management. On suspicion of PSC, medical evaluation and diagnosis must be promptly sought.

Currently, there is no specific cure for PSC, but some medications can help alleviate symptoms and slow disease progression. In addition to symptomatic treatments such as antibiotics for infections, vitamin supplements, and immunosuppressants to control inflammation, UDCA can also improve liver function and bile flow. Other new drugs are in clinical trials [98]. Although these medications can help manage PSC, liver transplantation remains the only effective treatment, especially for patients with advanced disease [98].

### 3.3. Fibrotic Process of BA and Current Treatment

Biliary atresia (BA) is a rare pediatric liver disease that emerges in the neonatal period. It is characterized by an immune-mediated obstruction of the bile ducts, both the extrahepatic and intrahepatic bile ducts, which disrupts the normal flow of bile. It is worth noting that newborns have a certain degree of immune tolerance, which means that their immune system usually does not produce a strong immune response to their own tissues at birth. However, this tolerance is not absolute. In some cases, the newborn’s immune system may be influenced by certain factors, leading to abnormal immune responses. Studies have shown that certain environmental factors, genetic susceptibility, or infections may trigger abnormal immune responses, leading to bile duct damage and fibrosis [92]. This might explain why biliary atresia occurs despite newborns typically having immune tolerance.

This obstruction leads to pathological jaundice, which, if left untreated, can progress to liver fibrosis, cirrhosis, and ultimately liver failure. These are serious, life-threatening conditions that can affect infants and young children [99]. In China, the incidence of BA is approximately 1 in 8000 live births, a rate comparable to the combined incidence of various childhood cancers. The initial treatment for BA involves a surgical procedure known as Kasai portoenterostomy, which aims to restore bile flow. However, if this intervention is unsuccessful, liver transplantation becomes the necessary and final treatment option to save the child’s life. It is important to note that early diagnosis and intervention are crucial for improving outcomes in BA [100].

Although existing animal experiments and clinical observations suggest that perinatal/neonatal viral infections, toxins, and genetic factors are important causes of BA, the specific etiology of BA remains unknown [101,102,103]. The pathogenesis of BA is not clear, but immune cells, such as natural killer (NK) cells, B cells, macrophages, and CD4^+^ and CD8^+^ T cells, are related to direct or indirect BEC damage, leading to bile duct injury [104]. Taking B cells and T cells as examples, BA, as an autoimmune disease, involves the expansion of B cells and T cells; these cells can recognize self-antigens and cause tissue damage, which is one of the pathogenic mechanisms of this disease. In the liver of children with BA, excess mature B cells not only produce pathogenic IgG autoantibodies but also act as antigen-presenting cells, thereby promoting the activation of cytotoxic T cells. Therefore, clearing excessive mature B cells can achieve a “two birds with one stone” effect [101]. The downregulation of regulatory T cells promotes bile duct injury mediated by Th1 cells in mouse BA induced by Ross River virus (RRV) [105]. The accumulation of neutrophils around the bile ducts may be related to the downregulation of STAT3 and the increased expression of chemokines [106]. In addition, in BA, *CD177*^+^ cells express interferon-stimulated and neutrophil degranulation genes, and high levels of mitochondria and reactive oxygen species (ROS) in *CD177*^+^ cells lead to the production of neutrophil extracellular traps (NETs) and result in bile duct cell death [107]. The hepatic artery accompanies and nourishes the bile ducts. When the hepatic artery is ischemic, the nutrition required for bile duct growth is limited, hypoxia-inducible factor alpha (HIF-α) is activated, and downstream such as cyclooxygenase 1/2 *(COX1/2)* are upregulated, causing bile duct injury; this further develops into bile duct stenosis or atresia, which may be one of the causes of the pathogenesis of BA. However, currently, there is almost no research on bile duct ischemia in BA, which may become a new research direction. In addition to viruses, inflammation, and immune responses being pathological mechanisms of BA, the destruction of the apical-basal polarization of BA bile duct cells [108], primary ciliary dyskinesia [109], the deposition of β-amyloid [100], changes in the ECM and ecological niche of bile duct cells [110], and the instability of the fate of bile duct cells [100,111] may also be newly uncovered pathological mechanisms of BA.

**Figure 3:**
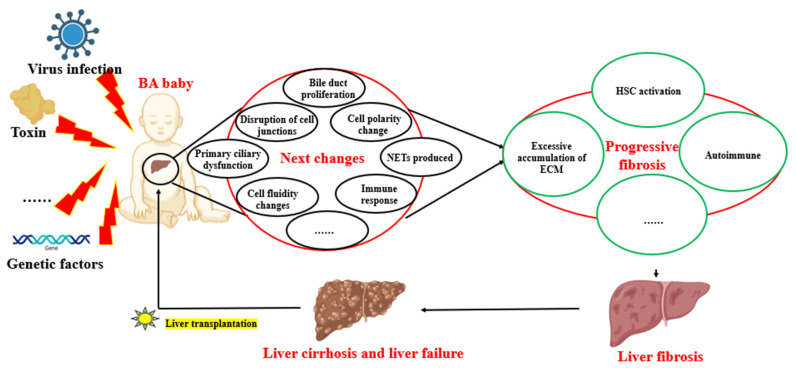
The pathogenetic and fibrotic mechanisms of BA. The etiology of BA may involve complex interactions among multiple factors, including viral infections, toxins, and genetic elements. Following BA, there is bile duct proliferation, disruption of cell junctions, altered cell polarity, and activation of the immune response. These changes lead to HSC activation, excessive ECM accumulation, and autoimmunity, ultimately resulting in liver fibrosis, cirrhosis, and liver failure, necessitating liver transplantation to extend the patient’s lifespan. BA: biliary atresia; HSCs: hepatic stellate cells; ECM: extracellular matrix. Diagrams were created with the help of BioRender software (© 2024 BioRender).

The pathogenesis of BA involves multiple immunopathological mechanisms. Recent studies have shed light on the potential factors contributing to the progression of liver fibrosis in BA. For instance, defects in B cell lymphangiogenesis and tolerance may lead to the expansion of self-reactive B cells and killer T cells, contributing to bile acid stasis. This stasis can inhibit the inflammatory response of hepatic macrophages and the scavenging function of Kupffer cells, while a deficiency in tissue-repairing CX3C chemokine receptor 1^+^ T/NK (*CX3CR1*^+^ T/NK) cells may disrupt fibrosis control mechanisms [101]. Recent studies have found that the glycodeoxycholic acid/sphingosine-1-phosphate receptor 2/Z-DNA binding protein 1/phosphorylated-mixed lineage kinase domain-like pseudokinase (GDCA/S1PR2/ZBP1/p-MLKL)-mediated necrotic apoptosis of macrophages plays a crucial role in the pathogenesis of BA liver fibrosis, and targeting this process may represent a potential therapeutic strategy for BA [112]. Additionally, an aberrant expression of SRY-box transcription factor 9 (SOX9) in hepatic progenitor cells (HPCs) has been implicated in the fibrotic process [113]. SOX9, which is regulated by the mechanosignaling factor yes-associated protein 1 (YAP1), plays a role in liver fibrosis, with its downregulation potentially improving the condition [114]. However, the mechanisms of the YAP1/SOX9 pathway in BA-induced liver fibrosis require further investigation. Epigenetic factors also appear to play a role, as histamine has been found to correlate positively with fibrosis severity, suggesting that it is a potential target for intervention [115]. Moreover, the long non-coding RNA (lncRNA) adducin 3 antisense RNA1 (ADD3-AS1) has been identified as a facilitator of HSC migration, indicating its potential as a diagnostic marker or therapeutic target [116]. Lastly, the downregulation of miR-145, which may lead to increased ADD3 expression, has been associated with the promotion of liver fibrosis in BA, highlighting the intricate network of genetic and molecular interactions involved in the disease’s progression. Understanding these pathways is crucial for developing targeted therapies for BA. Recent studies have highlighted the potential of targeting specific molecular pathways to attenuate BA fibrosis [117]. Qiu et al.’s research suggests that miR-145, along with transforming growth factor-beta/SMAD family member 2 (TGF-β/SMAD2) signaling, could serve as a pharmacological target to mitigate BA fibrosis [118]. Similarly, Xiao et al. found that the upregulation of miR-200b in BA patients enhances the proliferation and migration of HSCs via the phosphoinositide 3-kinase/protein kinase B (PI3K/AKT) pathway, indicating a possible epigenetic intervention point [119]. These findings underscore the significance of epigenetics in BA fibrosis research and potential therapeutic strategies. Furthermore, the role of bacterial lipopolysaccharides (LPSs) in inducing the epithelial-to-mesenchymal transition (EMT) and transforming biliary epithelium cells into myofibroblasts (MBs) has been established, which contributes to the progression of fibrosis by increasing collagen secretion and ECM deposition [120]. This process leads to liver parenchyma hypoxia and angiogenesis, exacerbating the condition. Additionally, serum protein extravasation, due to the immaturity and inflammatory nature of hyperpermeable neovascularization, further aggravates hepatic fibrosis [121]. These insights provide a clearer understanding of the complex mechanisms underlying BA fibrosis and open avenues for novel therapeutic interventions. Several pathogenetic and fibrotic mechanisms of BA are listed in Figure 3.

The Kasai procedure is the standard first-line treatment for BA, typically performed within 60 days of birth. This surgery involves attaching a loop of the intestine to the liver to create a pathway for bile flow [122]. Although the procedure can temporarily alleviate symptoms, most children will still require a liver transplant within a few years [123].

### 3.4. Similarities and Differences in Fibrosis Characteristics of PBC, PSC, and BA

PBC, PSC, and BA are all chronic cholestatic liver diseases, all involve autoimmune dysfunction and inflammation, and all lead to liver fibrosis. Their autoimmune mechanisms are different. PBC is mainly characterized by AMA and T cell-mediated immune responses [124,125]. PSC is closely associated with IBD, and abnormal immune responses lead to chronic inflammation and fibrosis. BA is a neonatal disease that may be triggered by a congenital immune response to viral infection, leading to bile duct obstruction and fibrosis. The fibrosis characteristics of these three diseases are also different. PBC causes chronic inflammation and destruction of the bile duct epithelial cells, leading to bile stasis and liver fibrosis [126]. As the disease progresses, fibrosis can spread throughout the liver, eventually leading to cirrhosis. In liver tissue, chronic inflammatory cell infiltration and fibrotic bands can be observed around the bile ducts [29,127]. PSC is characterized by segmental narrowing and dilation of the bile ducts. Fibrosis typically distributes along the bile ducts, forming an “onion-skin” pattern of fibrosis [127]. Fibrosis around the bile ducts and proliferation of the bile duct epithelium is accompanied by inflammatory cell infiltration within the bile ducts [84,127]. BA leads to bile duct atresia or underdevelopment, preventing bile from being excreted, which quickly causes intrahepatic bile stasis and fibrosis. Fibrosis progresses rapidly, usually leading to cirrhosis within a few months. In liver tissue, extensive cholestasis, ductal reaction, and fibrosis can be observed [101]. While the therapeutic targets for cholestatic diseases are indeed well defined, the treatment options become limited as the disease progresses to fibrosis. At this advanced stage, liver transplantation often emerges as the primary recourse due to the scarcity of effective pharmacological interventions. Current research is focused on developing new drugs and treatment strategies to offer alternatives before reaching the point where transplantation is the only option.

## 4. New Treatment Approaches in Regenerative Medicine

**Figure 4:**
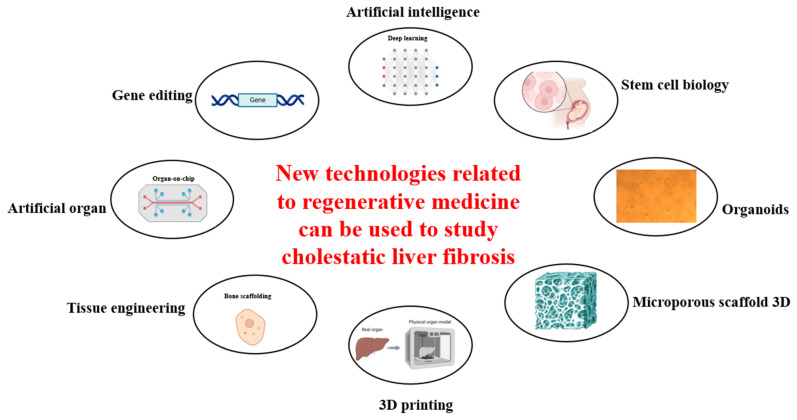
Some new technologies related to regenerative medicine can be used to study and aid in the treatment of cholestatic liver fibrosis. Diagrams were created with the help of BioRender software (© 2024 BioRender).

Regenerative medicine is a rapidly growing field that holds great promise in treating a variety of diseases, and indeed progressively transforming the landscape of treatments for liver fibrosis. Some new technologies related to regenerative medicine, as shown in Figure 4, can be used to study and aid in the treatment of cholestatic liver fibrosis. Recent advancements have shown that stem cell technology, combined with bioengineering and gene therapy, can create new platforms to potentially reverse liver failure and regenerate healthy cells. For instance, the development of bile duct cells from stem cells has opened new opportunities for understanding and treating liver diseases. Moreover, innovative therapies targeting the ECM are showing promise as alternatives to liver transplantation. These breakthroughs represent a significant leap forward in medical science, offering hope for more-effective and less-invasive treatments for liver conditions.

### 4.1. MSC Treatment

Mesenchymal stem cell (MSC) therapy is the first cutting-edge technology applied in regenerative medicine that aims to repair damaged tissues and organs. By utilizing the inherent abilities of MSCs to differentiate into various cell types and modulate immune responses, researchers and clinicians are exploring their potential in treating conditions ranging from osteoarthritis to cardiovascular diseases. The process involves harvesting MSCs from sources like bone marrow or adipose tissue, expanding them in a lab, and administering them to patients, offering hope for innovative treatments in the future. At present, the mechanism of MSC application in the treatment of cholestatic liver fibrosis mainly focuses on different aspects of pathogenesis by its function in immunomodulation, differentiation potential, anti-inflammatory, and anti-fibrotic effects: MSCs regulate immune response, inhibit the proliferation of T cells and B cells, and promote the differentiation of anti-inflammatory M2 macrophages by secreting biological factors and interacting with immunity [128,129]; MSCs can differentiate into hepatocytes and reconstruct the liver [130]; MSCs can secrete a variety of anti-inflammatory factors, inhibit inflammatory responses, and reduce hepatocytes and bile duct cells [131]; and MSC-derived exosomes and extracellular vesicles inhibit the progression of fibrosis [131,132]. Compared with induced pluripotent stem cells (iPSCs), MSCs generally have a lower risk of tumor formation, although their long-term safety still needs to be evaluated. Apart from that, healthy BECs can be derived from MSCs. Research indicates that MSCs have the capacity for multidirectional differentiation, capable of transforming into a variety of cell types, including BECs [133]. Utilizing MSC-derived BECs to improve cholestatic injury is a promising strategy. MSCs not only differentiate into the required cell types but also possess immunomodulatory and tissue repair capabilities, making them potentially powerful in treating cholestatic injuries.

Several studies on cholestatic liver disease and fibrosis are in the clinical trial stage, for example, an ongoing study at the People’s Hospital of Wuhan University in China evaluating the potential of MSCs for the treatment of liver cirrhosis [134]. The study has been approved by the China Medical Products Administration. The United Kingdom University of Birmingham is conducting a phase IIa clinical trial (NCT02997878) to evaluate the safety and drug activity of human umbilical cord blood-derived MSCs in patients with PSC. Although MSCs are well studied, there are still some shortcomings, such as low transplant survival, difficult migration, inconsistent treatment effects, and potential side effects such as fever. The future for the use of MSCs to treat cholestatic liver fibrosis appears promising, with several innovative strategies on the horizon. Genetically modifying MSCs to boost their anti-inflammatory and anti-fibrotic properties could offer a more potent treatment option. Additionally, the development of targeted delivery systems aims to enhance the efficacy of MSCs, ensuring they reach the affected liver areas more effectively. Personalized treatment plans based on the patient’s specific condition could optimize outcomes, while the use of MSCs as “seed cells” in conjunction with other bioengineering technologies may revolutionize tissue repair and regeneration in liver diseases.

### 4.2. iPSCs Differentiate into Hepatocyte-like Cells and Bile Duct Cells

Induced pluripotent stem cells (iPSCs) are a technology via which specific transcription factors in somatic cells are reprogramed to form pluripotent stem cells. iPSCs and MSCs share some similarities, including regenerative potential and immune modulation, but they also have many differences. Compared to MSCs, iPSCs can differentiate into almost all types of cells in the body, including nerve cells, cardiomyocytes, and liver cells. Because iPSCs can be generated from the patient’s own cells, they are less likely to cause immune rejection at the time of cell transplantation. iPSC technology does not involve the use of embryos, avoiding the ethical issues associated with embryonic stem cells. iPSCs have a wide range of applications in regenerative medicine, disease model construction, and drug screening, including for repairing or replacing damaged tissues and organs. By generating iPSCs for patients with specific diseases, disease models can be established that can be used to study the pathogenesis of diseases and screen potential drugs. By generating iPSCs from the patient’s own cells, it is possible to develop personalized treatment plans that reduce side effects and improve efficacy. At present, the research on iPSCs in cholestatic liver fibrosis mainly focuses on four aspects: liver and bile duct organoid models [135], drug screening [136], disease models [137], and cell therapy [136]. Although iPSC technology has great potential, it also faces some challenges and shortcomings: different iPSC cell lineages are heterogeneous, which may affect their consistency and reliability in clinical applications; iPSCs are also tumorigenic; the induction efficiency of iPSC is relatively low; and the cost of clinical use is high and difficult. Future research directions can focus on improving induction efficiency, expanding applications, and improving safety.

The use of iPSC-derived hepatocyte-like cells and bile duct cells in cholestasis is still in a primitive stage, and some studies have tested other iPSC-derived cells in human trails [138]. However, the research related to the cholestasis is still using animal models, such as in liver regeneration [139] and bile duct repair [140] and genetic disease modeling [141]. Although promising effects were observed, arriving at a clinical assessment still has a long way to go. Future research directions for iPSCs in cholestatic liver fibrosis may include the use of iPSC-derived hepatocyte-like cells and cholangiocytes to establish in vitro models of cholestatic liver fibrosis. These models can be used to study the pathogenesis and progression of the disease [142]. Additionally, iPSC-derived cells can be used for high-throughput drug screening to find potential therapeutic drugs. Drug toxicity can also be tested to assess safety [142]. By combining gene-editing technologies such as clustered regularly interspaced short palindromic repeats/CRISPR-associated protein 9 (CRISPR/Cas9), the role of specific genes in cholestatic liver fibrosis can be studied to explore new therapeutic targets [143]. iPSCs can be generated from a patient’s cells and differentiated into hepatocyte-like cells and cholangiocytes for personalized disease research and treatment plan design [143]. Finally, co-culturing iPSCs with other model liver cells can be used to study the effects of iPSCs on these cells to investigate the role of iPSCs.

### 4.3. Hepatic Organoid Culture and Application

Organoids are miniature tissue structures formed by in vitro three-dimensional (3D) cultures and are derived from stem cells or organ progenitor cells. These 3D cell aggregates are capable of self-organizing and differentiating into models that are structurally and functionally similar to their corresponding organs in the human [144]. The application of 3D organoids mainly focuses on disease model construction, drug screening, and toxicity evaluation. In terms of cholestasis-related animal study, the construction of a mouse model of bile duct ligation (BDL) at 4 weeks is an excellent model of cholestatic liver fibrosis [145]. Extracting the liver from BDL mice and culturing it into liver organoids would be an excellent ex vivo model. These organoids can be applied in drug screening and toxicity testing, improving the efficiency and accuracy of drug development. Imagine observing the differences between the liver organoids of BDL mice and normal mice under a microscope in terms of size and morphology—it would be fascinating. Additionally, by generating organoids from individual cells, personalized disease research and treatment plans can be designed, offering more precise medical services [146]. Organoids hold potential in regenerative medicine, as they can be used for tissue repair and regeneration, and may even be used for organ transplantation in the future [147].

Organoid technology has its own drawbacks and challenges. Most current organoids lack a vascular system, which limits their ability to mimic complex tissues and organ functions. Without vascularization, organoids have low efficiency in nutrient and waste exchange, affecting their long-term survival and function. The production process of organoids is complex and costly, making it difficult to achieve large-scale production. This limits its widespread use in high-throughput drug screening and clinical applications. At present, there is a lack of uniform standards and specifications for the culture and use of organoids, which makes it difficult to compare and validate results between different laboratories. Although organoids are able to mimic the basic structure and function of some organs, they still cannot fully reproduce the complexity of organs in vivo. For example, organoids often lack a complete immune system and nervous system. Furthermore, in vitro models such as spheroids and organoids often face challenges in replicating the complex bile flow found within the body. The lack of a structured and functional sinusoidal vascular network can hinder proper bile flow, leading to issues with nutrient and waste exchange. Effective communication between hepatocytes and BECs is crucial for maintaining liver function. In organoids, this communication may be disrupted due to the absence of a fully developed microenvironment and vascularization, potentially leading to unstable and immature cultures that may not accurately simulate in vivo conditions [148,149]. Despite these limitations, ongoing research and technological innovations continue to enhance the reliability of these models. The shortcomings and challenges need to be further explored and addressed in future research to improve the application potential and reliability of organoid technology. Future organoid research in the application mainly focusses on combining the technology with bioengineering, studying how to make organs’ multicellular cultures with a bioreactor to have vascularized, multi-organo-organoid interactions, and mimicking complex pathological mechanisms.

### 4.4. Future Perspective

Mesenchymal stem cells (MSCs) and induced pluripotent stem cells (iPSCs) are administered to patients through various methods, depending on the specific conditions being treated and the goals of therapy. For liver diseases, enhancing the survival and homing capabilities of MSCs is crucial to improve engraftment efficacy [150]. The current strategies, including hypoxic priming, drug pretreatment, gene modification, and cytokine pretreatment, as well as splenectomy and local irradiation, are used to improve MSC survival and homing capability, and enhance cell engraftment and therapeutic efficiency of MSC therapy [150]. The methods of MSC and iPSC administration to patients include intravenous infusion [151], intravascular administration [152], or direct injection into the target tissue [153]. The success of engraftment in the liver is evaluated by tracking the survival, proliferation, and functional integration of the transplanted cells. Techniques such as imaging, histological analysis, and measurement of liver function parameters are used to assess engraftment. Clinical trials have shown that MSC transplantation can restore liver function and alleviate liver damage, but the long-term efficacy and the optimization of cell homing still present challenges [154]. Strategies to enhance cell engraftment include preconditioning of cells, genetic modification, and the use of specific delivery vehicles or scaffolds to support cell survival and integration [155,156]. These approaches aim to overcome the limitations of engraftment and improve the therapeutic potential of MSC and iPSC therapies for liver diseases.

Other regenerative medicine methods, such as organ-on-chip technology, which replicates human physiology and diseases in vitro, has shown promise for precision medicine in cholestatic diseases [157]. These devices can mimic the intricate architecture and communication pathways of the liver and bile duct, offering a dynamic environment to study disease mechanisms and test therapeutic interventions [158]. The integration of multiple organ systems on a single chip further enhances the potential for personalized medicine approaches by providing a more systemic understanding of disease progression and treatment response.

The study of regenerative medicine in cholestatic liver fibrosis and liver transplantation is of great significance. Regenerative medicine, through technologies such as stem cell therapy and tissue engineering, is expected to provide alternative treatment options and alleviate the shortage of donors [159]. Regenerative medicine techniques, such as MSC treatment, can promote liver tissue regeneration, reduce liver fibrosis, and improve liver function and quality of life [160]. Regenerative medicine, through methods such as autologous stem cell transplantation, is expected to reduce immune rejection and postoperative complications [161]. Regenerative medicine research can reveal the pathological mechanism of cholestatic liver fibrosis and discover new therapeutic approaches [162]. Regenerative medicine can help to develop personalized treatment options and develop the most appropriate treatment strategies based on the specific condition and genetic characteristics of patients to improve treatment [163]. The advantages of regenerative medicine technology provide it with broad application prospects in disease research and treatment. Only by applying such new technologies can the new technologies of regenerative medicine benefit mankind and alleviate the suffering caused by diseases.

## 5. Discussion

Cholestasis not only harms the liver but also significantly impacts intestinal health. In fact, if the transportation of bile acids through the biliary ducts is impaired, one would expect a low concentration of bile acids in the bowel. Cholestasis leads to a reduction in bile salts in the small intestine, affecting the absorption of fats and fat-soluble vitamins (such as vitamins A, D, E, and K) [164]. This can result in steatorrhea, with foul-smelling stools. Due to the impaired absorption of fat-soluble vitamins, there may be a deficiency in vitamin K (leading to coagulation disorders), vitamin A (leading to night blindness), vitamin E (leading to cerebellar ataxia, peripheral neuropathy, and retinal degeneration), and vitamin D (leading to osteomalacia). The enterohepatic circulation of bile acids and the gut microbiota have a close interaction [165]. Bile acids can alter the composition of the gut microbiota, and conversely, the gut microbiota can affect the metabolism of bile acids. This interaction plays an important role in the pathogenesis of cholestatic liver diseases. Cholestasis may lead to intestinal inflammation, further damaging the intestinal mucosa and affecting intestinal health [166,167].

The most-used preclinical models currently for PBC/PSC/BA include genetically modified, chemically inducible, biologically inducible, and protein-immunized models, but they all have limitations that constrain further research and weaken their connection with clinical practice [168]. For example, dnTGF-βRII mice are commonly used in PBC research. These mice can perform causal analysis and elucidation of the key steps in the pathogenesis of PBC, allowing for a deeper understanding of the disease. However, a major drawback of these mice is that the immunological and pathological characteristics they exhibit have limited correlation with clinical practice [168]. Multidrug-resistant 2 deficient (*mdr2*^−/−^) mice are commonly used in PSC research. Since these mice are a model for cholestasis due to a lack of phospholipids in bile, they may not fully mimic all the physiological and pathological features of human PSC [169]. The RRV infection of newborn mice is commonly used in BA research, but the short survival time of the RRV model limits the study of the late-stage fibrosis process in BA [170]. The BDL model can be used to simulate cholestatic liver disease, but it primarily simulates damage to the large bile ducts, while autoimmune liver diseases often involve damage to the small bile ducts. Additionally, the BDL model cannot fully replicate the immune responses and pathological processes in human diseases, so it may not be precise enough when studying autoimmune mechanisms [145]. The field of regenerative medicine is exploring new methods to overcome the limitations of traditional animal models, such as organoid technology. The latest advancements in regenerative medicine provide new directions for research and hold the promise of improving the quality and effectiveness of clinical treatments.

Currently, there are indeed clinical trials underway that utilize regenerative medicine to treat cholestatic liver diseases. For instance, drugs based on FGF19 have shown promising results in the selective treatment of patients with PBC, PSC, NASH, or hepatocellular carcinoma (HCC) [171]. Additionally, several novel drugs are in development, including norUDCA [172] and Simtuzumab [173]. These studies and trials offer new hope and possibilities for the treatment of cholestatic liver diseases.

An increasing number of studies are using new regenerative medicine methods to explore the mechanisms of cholestatic liver diseases and fibrosis. Take organoids for instance; the cholangiocyte organoids cultured from liver biopsy specimens obtained from infants with BA and healthy individuals, as demonstrated by Amarachintha et al., revealed delayed epithelial development and barrier function in patients with BA [174]. Fotios et al. reconstructed the mouse extrahepatic biliary tree using primary human extrahepatic cholangiocyte organoids (ECOs), providing a proof of principle for organ regeneration using in vitro expanded primary human cholangiocytes [175]. Utilizing similar hepatobiliary system organoids, Ceilia et al. discovered that tumor necrosis factor-like weak inducer of apoptosis/fibroblast growth factor-inducible 14 (TWEAK/FN14) promotes the activation of the pro-fibrotic pathway in liver progenitor cells expressing Prominin-1 in bile acids [176]. Meng et al. demonstrated through the study of liver organoids and Klebsiella pneumoniae that Klebsiella pneumoniae plays a key role in post-Kasai cholangitis, and mediates the potential mechanism of liver fibrosis through the interleukin 13/transforming growth factor beta 1 (IL-13/TGF-β1) pathway [177]. All of this demonstrates the tremendous potential of new regenerative medicine methods in the study of disease mechanisms and the great interest in the effective application of new methods. Although current research has made certain breakthroughs in exploring the mechanisms of cholestatic liver disease and hepatic fibrosis, reflecting the advantages of new regenerative medicine methods, these methods are still imperfect and in the preliminary stages when it comes to investigating cholestatic liver disease and hepatic fibrosis, and much research and exploration are still needed. For example, the bile duct ligation (BDL) model can be used to study cholestatic liver fibrosis [145], although there is research related to stem cells [178]. However, there are still few studies on the new methods of regenerative medicine combined with the BDL model in the field of cholestatic liver fibrosis, and there is almost no research on 3D hepato-biliary-like organoids and 3D bioprinting in BDL. Moreover, there are many concerning issues with the new methods of regenerative medicine in exploring cholestatic liver fibrosis.

Firstly, the integration of new technologies in medical treatments presents a multifaceted challenge, particularly in the realm of safety and efficacy. The development of safety standards for emerging medical technologies is a complex process that involves extensive research, including basic experiments and clinical trials, to ensure that any adverse reactions are well understood and mitigated. In the specific case of MSC transplantation for cholestatic liver fibrosis, the timing and conditions under which this treatment is most effective are still under investigation. Clinical trials have shown promise in MSCs restoring liver function and reducing liver damage, but the optimal application stage for different liver conditions requires further study.

Moreover, the construction of organoids and organs via 3D bioprinting for regenerative medicine is a burgeoning field. These technologies hold the potential for organ repair and even creation, but the journey from laboratory to clinical application is fraught with challenges, including replicating the complex architecture and functionality of native tissues [179,180]. As research progresses, it is crucial to address these challenges through collaborative efforts between scientists, clinicians, and regulatory bodies to ensure that these innovative treatments can be safely and effectively integrated into patient care.

After 60 years of development, clinical allograft liver transplantation is well established and xenograft liver transplantation is just beginning [181,182]. Liver transplantation is indeed a critical intervention for patients with end-stage liver disease, offering a chance for extended life and improved quality of life. The challenges outlined, including donor organ scarcity, high costs, and surgical risks, are significant hurdles in the field of transplant medicine. Looking ahead, the integration of new technologies and innovative research approaches holds promise for addressing these issues. Advances in tissue engineering and regenerative medicine, for instance, may one day allow for the growth of liver tissues in the laboratory, reducing the dependency on donor organs. Additionally, precision medicine could tailor treatments to individual patients, potentially lowering the risks and improving outcomes. The pursuit of such advancements reflects the dynamic nature of medical science, driven by a commitment to enhancing patient care and expanding the horizons of what is medically possible.

## 6. Conclusions

In this review, we focus on the fibrosis progression of cholestatic liver diseases such as PBC, PSC, and BA, and summarize, analyze, and discuss the current status and advantages of regenerative medicine treatments. Our review comprehensively explores the molecular and cellular mechanisms of fibrosis in cholestatic liver diseases such as PBC, PSC, and BA. Our review highlights emerging therapies such as MSC therapy, iPSCs, and organoid technology, offering potential breakthroughs in managing cholestatic liver diseases. By focusing on mechanistic pathways and novel treatments, this review provides a forward-looking perspective on bridging the gap between basic science and clinical applications in liver fibrosis. By focusing on the latest research, our review may reveal new targets and enhance therapeutic efficacy, potentially leading to more effective management and even curative strategies for cholestatic liver diseases.

Cholestatic liver fibrosis represents a complex medical challenge, with its pathogenesis varying significantly among individuals, necessitating personalized diagnostic and therapeutic approaches. Conditions including primary PBC, PSC, and BA serve as key examples in understanding the disease’s multifaceted nature. Despite advancements, the current knowledge remains fragmented, highlighting the need for continued research into these conditions. Over the past five years, substantial progress has been made, yet the quest for a comprehensive understanding and effective management of cholestatic liver fibrosis continues. The prospects of exploring the nature and treatment of diseases through regenerative medicine are promising, as regenerative medicine holds great potential in treating cholestatic liver fibrosis.

## Data Availability

Not applicable.

## Abbreviations

ADD3-AS1: adducin 3 antisense RNA1; AE2: anion exchanger; AP1: adaptor protein-1; APCs: antigen-presenting cells; ALP: alkaline phosphatase; AMA: anti-mitochondrial antibodies; ANCAs: anti-neutrophil cytoplasmic antibodies; ANAs: anti-nuclear antibodies; AKT: protein kinase B; BA: biliary atresia; BDL: Bile duct ligation; BECs: biliary epithelial cells; Cas9: CRISPR-associated 9; CCL-2: chemokine ligand 2; CCR-2: chemokine receptor 2; CCL24: C-C motif chemokine ligand 24; CD: Crohn’s disease; COX1/2: cyclooxygenase 1/2; CRISPR: clustered regularly interspaced short palindromic repeats; CYP7A1: Cholesterol 7α-hydroxylase; CX3CR1: fractalkine receptor; 3D: three-dimension; DR: ductal reaction; ECMs: extracellular matrices; EHBDs: extrahepatic bile ducts; EpCAM: Epithelial cell adhesion molecule; EMT: epithelial-mesenchymal transition; FAR: fatty acid regulator; FGF1: fibroblast growth factor 1; FXR: farnesoid X receptor; GDCA: glycodeoxycholate; HSCs: hepatic stellate cells; HLAs: human leukocyte antigens; HIF-α: hypoxia-inducible factor alpha; IBD: inflammatory bowel disease; IHBDs: intrahepatic bile ducts; IL-6: interleukin 6; iPSC: induced pluripotent stem cell; LPS: lipopolysaccharides; MB: myofibroblasts; MadCAM-1: mucosal addressin cell adhesion molecule-1; MLN: mesenteric lymph nodes; MSCs: mesenchymal stem cells; NASH: nonalcoholic steatohepatitis; NAFLD: non-alcoholic fatty liver disease; NK cells: natural killer cells; NETs: neutrophil extracellular traps; OCA: obeticholic acid; PBC: primary biliary cholangitis; PSC: primary sclerosing cholangitis; PDC-E2: pyruvate dehydrogenase complex-E2; PDGF: platelet-derived growth factor; PAMPs: pathogen-associated molecular patterns; p-MLKL: phosphorylated-mixed lineage kinase domain-like pseudokinase; PI3K: phosphoinositide 3-kinase; P4HA2: prolyl-4-hydroxylase alpha subunit 2; ROS: reactive oxygen species; Slit2-Robo1: Slit guidance ligand 2-roundabout guidance receptor 1; S1PR2: sphingosine 1-phosphate receptor 2; SMAD2: SMAD family member 2; STAT3: signal transducer and activator of transcription 3; SOX9: sex-determining region Y-box 9; SHP: Small Heterodimer Partner; TGF-β: Transforming growth factor beta; TFR: T follicular regulatory T cells; TCR: T cell receptors; TGR5: Takeda G protein-coupled receptor 5; TGF-β1: transforming growth factor-beta1; UC: ulcerative colitis; UDCA: ursodeoxycholic acid; VCAM-1: vascular cellular adhesion molecule-1; YAP1: Yes-associated protein 1; ZBP1: Z-DNA-binding protein 1.
